# Case Report: A female case of X-linked intellectual disability syndrome type 34 caused by a NONO frameshift variant and literature review

**DOI:** 10.3389/fped.2026.1739490

**Published:** 2026-02-05

**Authors:** Yige Zhang, Xiaoyan Shi, Xiao Xiao, Shuhua Yuan, Jihong Tang

**Affiliations:** 1 Department of Neurology, Children’s Hospital of Soochow University, Suzhou, China; 2 Department of Child Health Care, Linyi People’s Hospital, Linyi, China

**Keywords:** female patient, frameshift variant, NONO gene, X-chromosome inactivation, X-linked intellectual disability syndrome type 34

## Abstract

**Introduction:**

To characterize the clinical and genetic features of a female infant with X-linked intellectual disability syndrome type 34 (MRXS34) caused by a *de novo* NONO frameshift variant, expanding the understanding of phenotypic mechanisms in females for this X-linked disorder.

**Methods:**

Retrospective study of the clinical data of a 10-month-old female infant diagnosed with MRXS34 due to NONO gene variation in June 2024, along with a literature review.

**Results:**

The proband presented with global developmental delay, relative macrocephaly, generalized hypotonia, cardiac anomalies (patent foramen ovale, moderate tricuspid regurgitation, pulmonary hypertension), etc. Whole-exome sequencing (WES) identified a *de novo* heterozygous frameshift variant in NONO (NM_007363.5): c.994del (p.Gln322Lysfs*31), confirmed absent in both parents by Sanger sequencing. X-chromosome inactivation (XCI) analysis revealed extreme skewing (99% inactivation of the paternal X-chromosome). Transcriptome sequencing demonstrated significantly reduced NONO expression (TPM = 20.70 vs. controls 52.34 ± 5.81). Literature review encompassing 27 postnatal MRXS34 cases (all male) consistently reported intellectual disability/developmental delay (100%), craniofacial dysmorphism (100%), cardiac defects (91.3%, predominantly left ventricular non-compaction), and corpus callosum abnormalities (85%).

**Discussion:**

We report the first molecularly confirmed female MRXS34 patient. Her full phenotypic manifestation is attributed to the *de novo* NONO loss-of-function variant combined with extreme non-random XCI. This case critically expands the clinical spectrum of MRXS34, underscores the diagnostic importance of XCI analysis in females with XLID phenotypes, and provides insights into the mechanisms enabling female expression of X-linked recessive disorders.

## 1 Introduction

X-linked intellectual disability (XLID) constitutes a significant subset of inherited neurodevelopmental disorders, resulting from pathogenic variants in genes located on the X-chromosome, and it accounts for approximately 10% of inherited intellectual disability cases (1). Over 140 XLID-associated genes have been identified to date, including *DDX3X*, *USP9X*, *KDM6A*, *KDM5C*, *SMC1A*, *HDAC8*, *PCDH19*, *MECP2*, *FMR1*, and *CDKL5*. Males are predominantly and severely affected due to hemizygosity for X-linked genes. Female carriers, benefiting from random X-chromosome inactivation (XCI), are typically asymptomatic or exhibit milder phenotypes. However, *de novo* pathogenic variants combined with skewed XCI (SXCI), where the X chromosome carrying the wild-type allele is predominantly inactivated, can disrupt this protective mechanism, leading to full phenotypic expression in females (2, 3). Recently, several X-linked disorders that disproportionately affect females have been described, illustrating diverse inheritance modes such as semi-dominant (e.g., *DDX3X*), co-existing recessive and dominant patterns (e.g., *USP9X*), and unique phenotypes resulting from cellular mosaicism (e.g., *PCDH19*). These conditions highlight the expanding spectrum of X-linked disorders with female-predominant presentation, complex inheritance patterns, and notable sex differences (4).

The *NONO* gene (OMIM *300084), located at Xq13.1, encodes a multifunctional nuclear protein belonging to the Drosophila Behavior Human Splicing (DBHS) family. *NONO* plays crucial roles in transcriptional regulation, RNA processing (including splicing, stability, and nuclear retention), and DNA repair (5). *NONO* is also essential for neurodevelopment: studies in mouse models demonstrate that its deficiency impairs TET1 chromatin association and neuronal differentiation (6), and disrupts cortical neuronal migration and postnatal maturation (7). These mechanisms likely underlie the synaptic and cognitive deficits observed in humans with *NONO*-related disorders. Pathogenic variants in *NONO* cause MRXS34 (OMIM #300967), characterized by intellectual disability/global developmental delay, distinctive craniofacial dysmorphism (e.g., relative macrocephaly, long face, micrognathia), cardiac defects (notably left ventricular non-compaction—LVNC, septal defects), and structural brain abnormalities. Since its initial description in 2015, all 27 reported postnatal MRXS34 cases have been male (8–12). A single female fetus with a *NONO* missense variant was prenatally diagnosed but not molecularly confirmed postnatally.

Here, we present the first molecularly confirmed female patient with MRXS34 harboring a *de novo NONO* frameshift variant. Extreme skewing of XCI and the significantly reduced expression *NONO* gene confirmed by RNA-Seq, leading to the characteristic neurodevelopmental and cardiac phenotype. This case expands the known genotype-phenotype spectrum of MRXS34, provides evidence for the role of non-random XCI in female expression of XLID disorders, and offers critical insights for the diagnosis of X-linked recessive conditions in females.

## 2 Materials and methods

### 2.1 Patient

A 10-month-old Chinese female infant presenting with global developmental delay was evaluated at the Department of Pediatrics, Linyi People's Hospital, in June 2024, with comprehensive clinical data collected, including developmental assessments using the Gesell Developmental Schedules (GDS) at 10 months and 1 year 9 months, behavioral screening via the Autism Behavior Checklist (ABC Scale), neurophysiological evaluation through electroencephalography (EEG), neuroimaging via brain Magnetic Resonance Imaging (MRI) using a 3.0T Siemens Skyra scanner, cardiac assessment by transthoracic echocardiography, functional mobility evaluation using the Gross Motor Function Measure-88 (GMFM-88) during rehabilitation, and laboratory investigations such as blood and urine metabolic screening, thyroid function tests, and other relevant biochemical assays to exclude common genetic metabolic disorders. The study protocol was approved by the Institutional Ethics Committee of Linyi People's Hospital (Approval No. 202404-H-008), and written informed consent was obtained from the parents for genetic testing, publication of clinical data, and images.

### 2.2 Genetic analysis

Peripheral venous blood (EDTA-anticoagulated) was collected from the proband and their parents. Genomic DNA was extracted using the TIANamp Blood DNA Kit, with quality assessment confirming concentrations ≥20 ng/μL and optimal purity (A260/280 ratio 1.8–2.0). Whole-exome sequencing was performed using the xGen Exome Research Panel v2.0 for target capture (>39 Mb coding regions) and sequenced on the DNBSEQ-T7 platform (PE150, average depth ≥100x, coverage ≥98.5%). Bioinformatics analysis included quality control (Fastp v0.23.2), alignment to GRCh37/hg19 (BWA-MEM v0.7.12), variant calling (GATK HaplotypeCaller for SNVs/InDels; XHMM for CNVs), and annotation/filtering using SnpEff/SnpSift with population (gnomAD v2.1/v4.0), and disease databases (OMIM, HGMD, ClinVar). Variants were classified per ACMG/AMP 2015 guidelines with HPO-based phenotype correlation. Candidate variants underwent Sanger sequencing validation (ABI 3500xL) and methylation-specific MLPA excluded Silver-Russell syndrome (ME030-C3 probe mix for 11p15/UPD7/UPD14). X-chromosome inactivation analysis used HpaII digestion/capillary electrophoresis (ABI 3130xl), with extreme skewing defined as CR ≥ 3 (≥75% allele inactivation). RNA-seq quantified *NONO* gene expression level vs. controls, completing a multifaceted molecular investigation of this developmental delay case.

### 2.3 Literature review

A systematic literature search was conducted using PubMed, China National Knowledge Infrastructure (CNKI), and Wanfang Data databases. Search terms included “*NONO*”, “MRXS34”, “X-linked intellectual disability syndrome 34”, and “*NONO*-related intellectual disability”. The search covered publications from the inception of the databases until June 30, 2025. Only molecularly confirmed cases with available clinical data were included for phenotypic and genotypic analysis.

## 3 Results

### 3.1 Clinical features

The proband, a 10-month-old female, presented to Linyi People's Hospital in June 2024 with a primary complaint of “motor developmental delay for over 2 months.” She was the fourth pregnancy (G4) and third live birth (P3), delivered via full-term cesarean section. Her birth weight was 3.00 kg (appropriate for gestational age), and Apgar scores were 10 at 1, 5, and 10 min. During hospitalization for neonatal pneumonia, echocardiography revealed a patent foramen ovale (PFO, 2 mm), moderate tricuspid regurgitation, and pulmonary hypertension (systolic pulmonary artery pressure, sPAP 55 mmHg). Family history revealed idiopathic scoliosis in her 10-year-old elder sister. Her parents and two elder sisters (aged 10 and 6 years) exhibited no neurodevelopmental abnormalities. The first pregnancy ended in elective termination for social reasons. There was no history of consanguinity or significant teratogen exposure during pregnancy.

**Figure 1:**
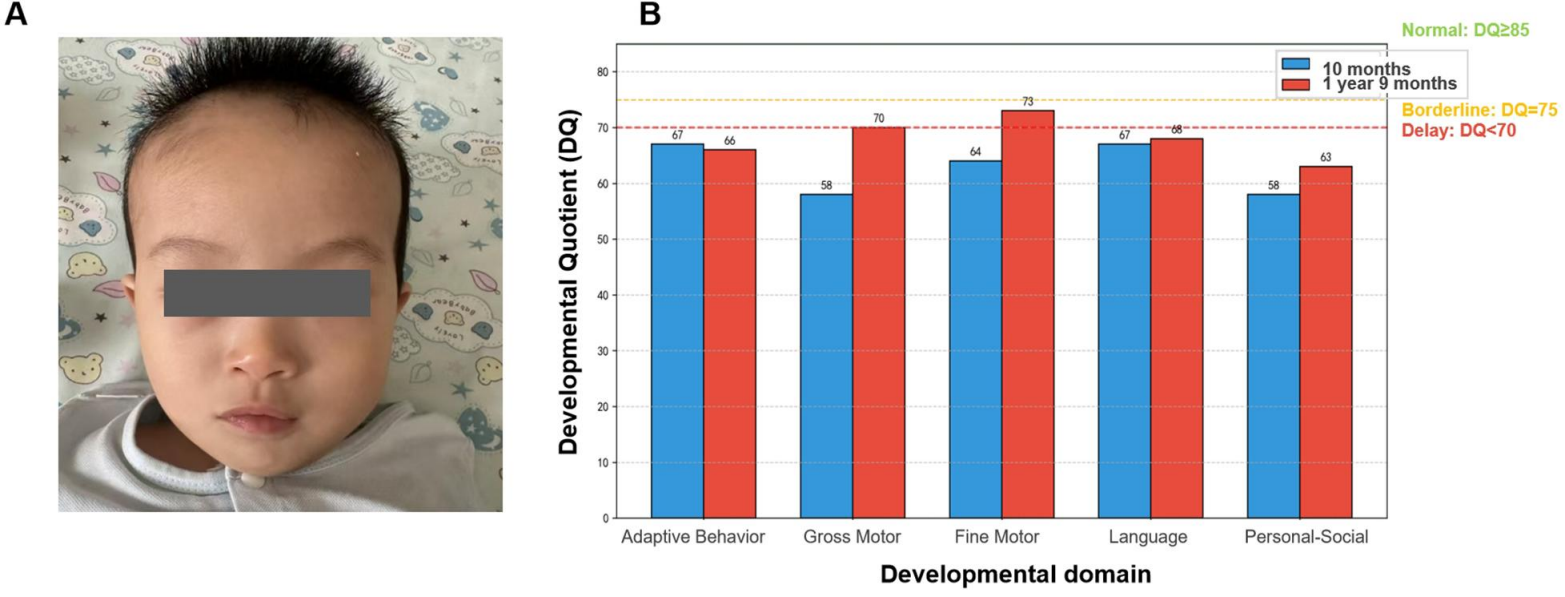
**(A)** craniofacial features of the proband at 10 months, showing triangular face, microtia, esotropia, low nasal bridge, flared nostrils, and micrognathia. **(B)** Gesell developmental quotients (DQ) assessed at 10 months and 1 year 9 months, demonstrating global delay across all domains.

At 10 months, physical examination revealed significant growth abnormalities with weight at 7.25 kg (<3rd percentile), length 70 cm (25th percentile), and macrocephaly (OFC 47.5 cm, >97th percentile; OFC/length Z-score +2.1). The infant displayed fair mental responsiveness but poor nutritional status, with normal cardiac findings. Distinct craniofacial dysmorphism included a triangular face, microtia with accessory auricle, left esotropia (passed basic vision screening), low nasal bridge with flared nostrils, and micrognathia (Figure 1A). Neurologically, generalized hypotonia (modified Ashworth scale grade 1) and severe motor delay were evident—unable to sit unsupported (normally achieved by 6 months) or maintain quadrupedal support (normally by 8 months), with absent parachute reflex. Gesell Developmental Schedules at 10 months confirmed global delay (DQ 58–67), showing 3–6 month lags across domains: Adaptive Behavior (67 points, 22-week developmental age), Gross Motor (58 points, 19 weeks), Fine Motor (64 points, 21 weeks), Language (67 points, 22 weeks), and Personal-Social (58 points, 19 weeks) (Figure 1B). Investigations included EEG (mildly slowed background without epileptiform discharges), cranial MRI (no abnormalities were found, but mild abnormal signals in the white matter of the bilateral frontoparietal and temporal lobes were observed, suggesting possible microscopic white matter changes), and normal metabolic/thyroid laboratory results, excluding common inborn errors.

### 3.2 Genetic findings

**Figure 2:**
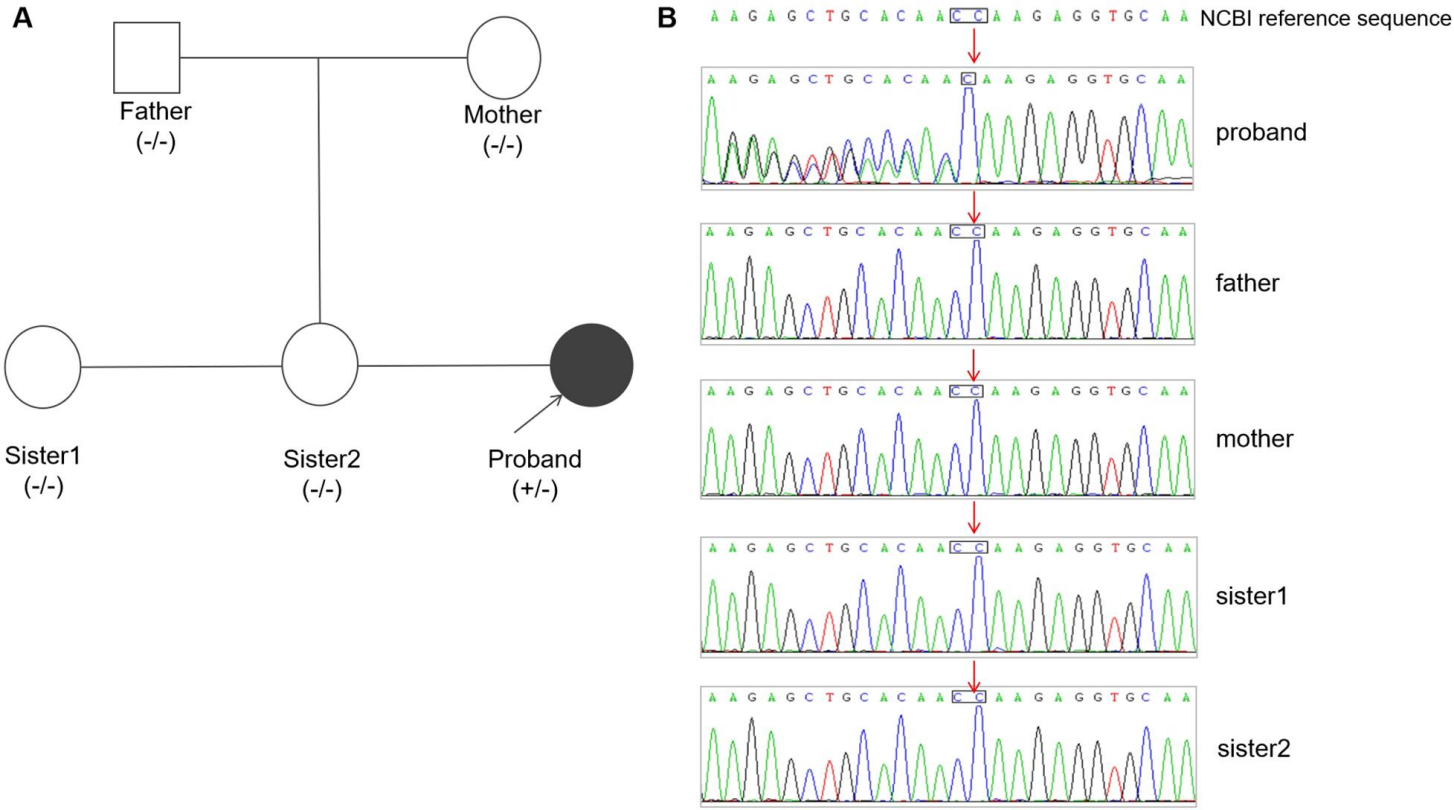
**(A)** pedigree of the proband's family. Arrow indicates the proband. Circles represent females; squares represent males; filled symbol indicates affected status. **(B)** Sanger sequencing chromatograms confirming the heterozygous c.994del variant (red arrow) in the proband and its absence in both parents and her sisters.

**Figure 3:**
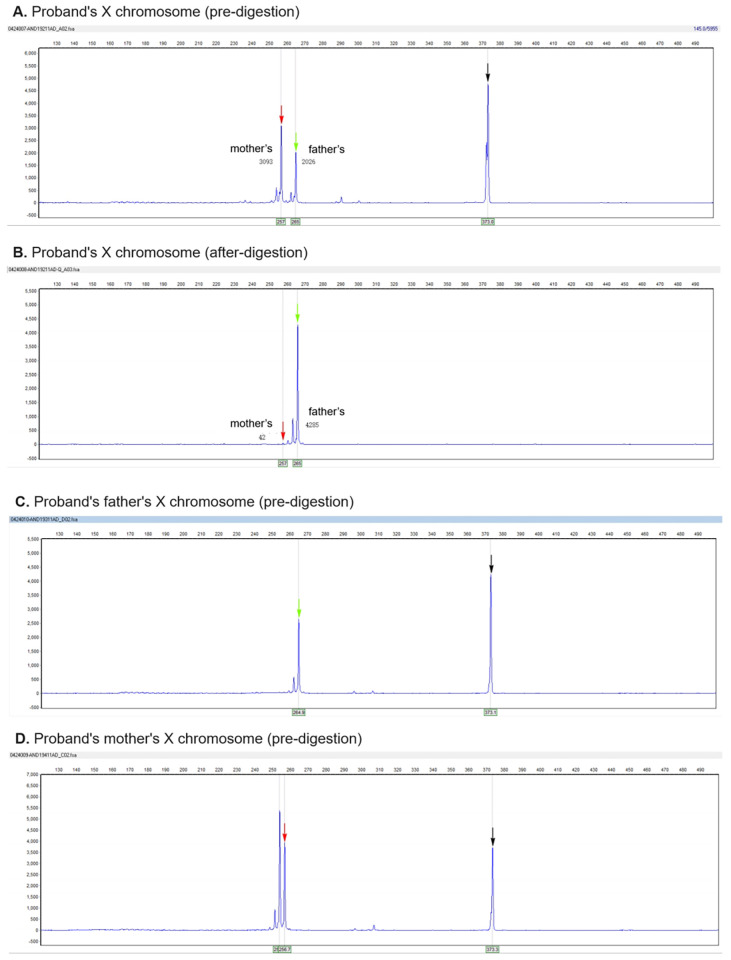
XCI analysis demonstrating extreme paternal X-chromosome skewing (99% inactivation). The electropherogram shows the amplification products of the polymorphic androgen receptor (AR) locus after HpaII digestion. The black arrow indicates the amplification product of the reference gene control (undigested). Complete digestion is confirmed by the absence of this peak in the digested samples. The numbers represent fragment length (bp) and peak height/area. Analysis reveals a near-complete predominance of one allele (paternal, representing the mutant chromosome), indicating non-random XCI with a calculated inactivation ratio of approximately 99:1 (CR >> 3).

Whole-exome sequencing (WES) identified a novel heterozygous frameshift variant in *NONO* exon 10 (NM_007363.5: c.994del, p.Gln322Lysfs*31), which Sanger sequencing confirmed as a *de novo* mutation absent in both parents and healthy sisters (Figures 2A,B). Following ACMG/AMP guidelines, the variant was classified as Pathogenic (PVS1 [null variant in the critical DBHS dimerization domain, likely triggering nonsense-mediated decay (NMD)] + PS2 [*de novo* occurrence] + PM2_Supporting [absent from population databases) (22). X-chromosome inactivation (XCI) analysis revealed extreme skewing (99% inactivation of the paternal X chromosome), favoring expression of the maternal X chromosome harboring the mutant *NONO* allele (Figure 3). RNA-seq confirmed significantly reduced *NONO* expression in the proband (TPM = 20.70 vs. controls: 52.34 ± 5.81, *p* < 0.001), consistent with NMD-mediated degradation. Methylation-specific MLPA ruled out Silver-Russell syndrome (normal 11p15/UPD7/UPD14). The combined evidence—clinical features (developmental delay, macrocephaly, hypotonia), brain MRI abnormalities, pathogenic *NONO* variant, skewed XCI, and reduced expression—confirmed the diagnosis of *NONO*-related X-linked intellectual disability syndrome (MRXS34).

### 3.3 Follow-up

After diagnosis, the child underwent comprehensive rehabilitation, including neurotrophic support (mouse nerve growth factor), hyperbaric oxygen therapy, pediatric tuina massage, joint mobilization, and hand function training, which continued post-discharge. At the most recent follow-up (age 1 year 9 months), growth parameters showed length at 77.0 cm (2.3rd percentile), weight at 8.35 kg (2.3rd percentile), and stable macrocephaly (head circumference 47.5 cm, >97th percentile). Motor milestones progressed with sitting achieved at 11 months, crawling at 14 months, and independent (though unsteady) walking by 1 year 8 months. Language development remained delayed, limited to reduplicated syllables (“baba,” “mama”) without communicative intent, with an overall developmental age equivalent to 12 months. Cardiac re-evaluation demonstrated improvement, with closure of the PFO, reduction of tricuspid regurgitation to mild, and resolution of pulmonary hypertension. Despite persistent global developmental delay, Gesell assessment revealed modest improvements in DQ scores compared to initial evaluation (Figure 1B), reflecting incremental progress through ongoing rehabilitation.

### 3.4 Literature review

**Figure 4:**
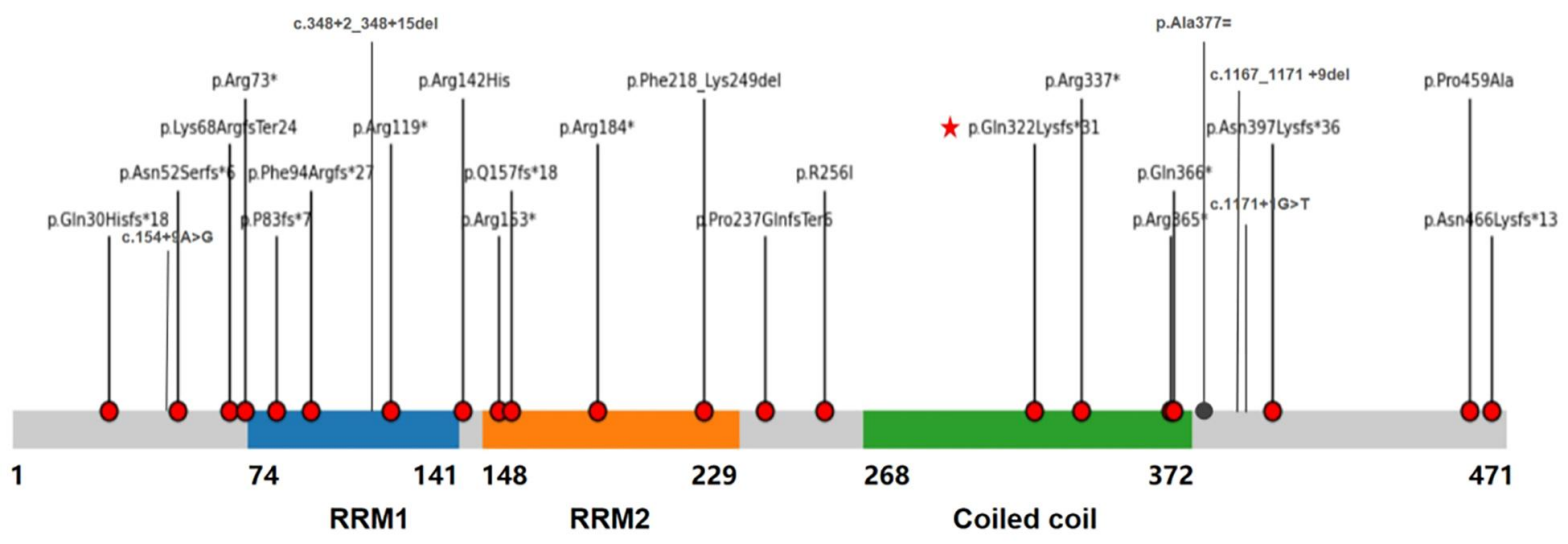
Schematic representation of the *NONO* protein structure (NP_009294.1) and distribution of reported pathogenic/likely pathogenic variants in MRXS34. Domains: RRM1 (RNA Recognition Motif 1, aa 1-93), RRM2 (aa 182-271), NOPS (aa 336-407), Coiled-coil (CC, aa 457-471). Variant types: Nonsense (Red squares), Frameshift (Blue triangles), Splice site (Green circles), Missense (Purple diamonds), Deletion (Orange bar). The red asterisk (*) indicates the novel frameshift variant (c.994del, p.Gln322Lysfs*31) identified in this female proband, located within the NOPS domain critical for dimerization. Variants are distributed across the entire gene length, with a high prevalence (85.7%) of loss-of-function (LOF) types.

A systematic review identified 16 publications (including one domestic report) documenting 27 live-born molecularly confirmed MRXS34 patients (all male) and 11 prenatally diagnosed cases (10 male, 1 female fetus) (8–11). Among live-born males, key clinical features included universal neurodevelopmental impairment (100% GDD/ID), consistent craniofacial dysmorphism (macrocephaly, long face, micrognathia; 100%), frequent cardiac defects [91.3%, predominantly LVNC (52.3%) followed by ASD/VSD], and prevalent brain anomalies (85% corpus callosum abnormalities). Additional findings involved hypotonia, seizures (∼7%), and skeletal/ocular abnormalities. Prenatal cases uniformly presented with severe cardiac malformations (LVNC, HLHS, Ebstein anomaly) often accompanied by other structural defects, demonstrating poor prognosis. Genetic analysis of all 38 cases revealed 28 distinct *NONO* variants, with 85.7% being loss-of-function (nonsense/frameshift/splice-site) distributed across all functional domains (Table 1 and Figure 4). Inheritance was primarily *de novo* (15/27 live-born) or maternal (11/27), while the sole reported female fetus carried a *de novo* VUS missense variant (p.Arg142His). Our female proband represents the first live-born female case with a pathogenic *NONO* variant, expanding the phenotypic and genotypic spectrum of MRXS34.

**Table 1:** Clinical phenotypes and genetic variations in reported MRXS34 patients caused by *NONO* pathogenic/likely pathogenic variants.

No.	Reference	Sex	Age	Variant	Inheritance	GDD/ID	Barin MRI	Craniofacial	Cardiac Abnomalities	Prenatal History
1	This study	F	10ms	c.994del, p.Gln322Lysfs*31	*de novo*	Yes	Abnormal signals in bilateral frontoparietal and temporal white matter	Yes	PFO, tricuspid regurgitation	None
2	(8)	M	17ys	c.1131G > A, Ala377=	*de novo*	Yes	Thickened corpus callosum, cerebellar hypoplasia	Yes	-	-
3	(8)	M	15ys	c.1394dup, p.Asn466Lysfs*13	Maternal	Yes	Thickened corpus callosum, Chiari type I malformation	Yes	-	Polyhydramnios
4	(8)	M	20ys	c.1093C > T, p.Arg365*	*de novo*	Yes	Agenesis of corpus callosum, ventricular enlargement	Yes	-	IUGR
5	(9)	M	17ys	c.1171 + 1G > T	*de novo*	Yes	Short and hypoplastic corpus callosum	Yes	LVNC	-
6	(29)	M	10ys	c.1093C > T, p.Arg365*	*de novo*	Yes	Agenesis of corpus callosum	Yes	LVNC, ASD, VSD, PDA, RVH	-
7	(29)	M	5ys	c.1394dup, p.Asn466Lysfs*13	*de novo*	Yes	Normal (at 3 months)	Yes	LVNC, ASD, VSD, PDA	-
8	(29)	M	15ms	Exons1-6 deletion	Maternal	Yes	Agenesis of corpus callosum	Yes	LVNC, PFO	-
9	(30)	M	2ys	c.154 + 5_154 + 6del, p.Asn52Serfs*6	Maternal	Yes	Thickened corpus callosum, Chiari type I malformation	Yes	LVNC, PFO, Ebstein anomaly	-
10	(21)	M	2ys	c.550C > T, p.(Arg184*)	Maternal	Yes	Normal	Yes	LVNC, ASD, VSD, PDA	-
11	(21)	M	4ys	c.1171 + 1G > T	*de novo*	Yes	Agenesis of corpus callosum	Yes	LVNC	-
12	(31)	M	10ms	c.1167_1171 + 9del	Maternal	Yes	-	Yes	LVNC, VSD, Ebstein anomaly	-
13	(32)	M	infant	c.767G > T, p.R256I	Maternal	-	Hydrocephalus	-	LVNC, tricuspid dysplasia	-
14	(28)	M	78ds	c.217C > T, p.(Arg73*)	*de novo*	Yes	Agenesis of corpus callosum (deceased)	Yes	NCC, ASD, PDA, right aortic arch	Cardiac
15	(28)	M	6ys	c.1009C > T, p.(Arg337*)	*de novo*	Yes	Agenesis of corpus callosum, ventricular enlargement	Yes	NCC, ASD, right aortic arch	Ebstein
16	(28)	M	1ys	c.90_114del, p.(Gln30Hisfs*18)	*de novo*	Yes	Hypoplasia of corpus callosum, ventricular enlargement	Yes	NCC	Brain MRI
17	(28)	M	20ys	c.1190_1191del, p.(Asn397Lysfs*36)	Maternal	Yes	Normal	Yes	-	Normal
18	(28)	M	10ms	c.651-1G > C, p.Phe218_Lys249del	Maternal	Yes	Agenesis of corpus callosum	Yes	NCC, ASD,	Cardiac, brain
19	(28)	M	29ys	c.217C > T, p.(Arg73*)	*de novo*	Yes	Abnormal corpus callosum	Yes	NCC, PDA	-
20	(20)	M	2ys	c.1375C > G, p.(Pro459Ala)	Maternal	Yes	-	-	VT, dilated cardiomyopathy	-
21	(20)	M	4ys	c.1375C > G, p.(Pro459Ala)	Maternal	Yes	-	-	None	-
22	(20)	M	4ys	c.1375C > G, p.(Pro459Ala)	Maternal	Yes	-	-	None	-
23	(20)	M	17ys	c.348 + 2_ 348 + 15del	*de novo*	Yes	Agenesis of corpus callosum	Yes	ASD, VSD, Ebstein anomaly	-
24	（9）	M	3ys	c.457C > T, p.(Arg153*)	*de novo*	Yes	-	-	Yes	-
25	(11)	M	infant	c.1131G > A, Ala377=	*de novo*	Yes	-	-	Cardiomegaly	-
26	(10)	M	-	c.279_282del, p.(Phe94Argfs∗27)	Maternal	Yes	Agenesis of corpus callosum	Yes	Yes	None
27	(10)	M	-	c.201_202dup, p.(Lys68ArgfsTer24)	-	Yes	Short corpus callosum	Yes	LVNC	None
28	(10)	M	-	c.710del, p.(Pro237GlnfsTer6)	*de novo*	Yes	Agenesis of corpus callosum	Yes	LVNC	None
Total	Live birth	28	0-29ys			26	17/20	21	21/23	
Fetus	(21)	M	1fetus	c.457C > T, p.(Arg153*)	*de novo*	-	-	-	Cardiomegaly, pulmonary stenosis	Renal, Cardiac
Fetus	(33)	M	3fetus	c.246_249del, p.P83fs*7	Maternal	-	-	-	LVNC, A/VSD, pulmonary stenosis	Cardiac
Fetus	(33)	M	2fetus	c.471del, p.Q157fs*18	Maternal	-	-	-	LVNC, A/VSD, pulmonary stenosis	Cardiac
Fetus	(34)	M	1fetus	c.154 + 9A > G	Maternal	-	-	-	HLHS	Cardiac
Fetus	(35)	M	1fetus	3’UTR deletion	*de novo*	-	Agenesis of corpus callosum	-	tricuspid regurgitation, Ebstein	Cardiac, brain
Fetus	(27)	M	1fetus	c.1096C > T, p.(Gln366*)	*de novo*	-	-	-	NCC, PDA, PAH, PFO	Cardiac
Fetus	(27)	F	1fetus	c.425G > A, p.(Arg142His)	*de novo*	-	-	Yes	aortic arch anomaly	Cardiac
Fetus	(36)	M	1fetus	c.355C > T, p.(Arg119*)	*de novo*	-	-	Yes	Cardiomy hypertrophy	Renal
Total	Fetus	11	Prenatal			-	1	2	11	Renal 2 cases

GDD, global developmental delay; ID, intellectual disability; MRI, magnetic resonance imaging; IUGR, intrauterine growth restriction; LVNC, left ventricular noncompaction; ASD, atrial septal defect; VSD, ventricular septal defect; PDA, patent ductus arteriosus; PFO, patent foramen ovale; RVH, right ventricular hypertrophy; HLHS, hypoplastic left heart syndrome; NCC, noncompaction cardiomyopathy; VT, ventricular tachycardia; PAH, pulmonary arterial hypertension; “-” indicates not mentioned or described in the literature.

## 4 Discussion

This study presents the first molecularly confirmed female patient with MRXS34, resulting from a *de novo NONO* frameshift variant (c.994del, p.Gln322Lysfs*31) and extreme skewing of XCI (99% paternal inactivation). This mechanism effectively created a functional hemizygous state for the mutant allele, overriding the typical protective effect of random XCI in female carriers of X-linked recessive disorders (2, 3). Her phenotype—global developmental delay, relative macrocephaly, hypotonia, PFO, tricuspid regurgitation, and white matter abnormalities—aligns remarkably well with the core features observed in affected males (8–11), although her cardiac manifestations (PFO, resolving TR) were milder than the prevalent LVNC in males. This case critically expands the clinical spectrum of MRXS34 and provides a paradigm for understanding female expression in XLID disorders.

*NONO*, a core member of the DBHS protein family (alongside SFPQ and PSPC1), functions as a multifunctional nuclear scaffold protein (13). Its domains (RRM1, RRM2, NOPS, Coiled-coil) facilitate interactions with DNA, RNA, and other proteins, enabling roles in transcriptional regulation, RNA processing (splicing, nuclear retention), DNA repair, and paraspeckle formation (5). *NONO* is crucial for neurodevelopment: knockout mice exhibit cerebellar defects, impaired synaptic function (particularly involving GABAergic transmission via genes like *GABRA2*), spatial memory deficits, and anxiety-like behaviors, mirroring the human intellectual disability phenotype (8). In cardiac development, *NONO* deficiency disrupts cardiomyocyte differentiation and promotes fibroblast proliferation, leading to LVNC and other structural defects (14). Its recent identification as a key candidate for X-linked contributions to congenital heart disease (CHD) gender differences further underscores its systemic importance (15). Additional roles in circadian rhythm regulation, metabolic gene coordination, DNA damage response via R-loop modulation, and myogenesis, highlight the pleiotropic effects of *NONO* dysfunction, explaining the multi-system involvement in MRXS34 (neurodevelopmental, cardiac, potential musculoskeletal) (16–18). The truncating variant reported here (p.Gln322Lysfs*31), located within the essential NOPS dimerization domain, is predicted to undergo NMD, resulting in haploinsufficiency, consistent with the observed significant reduction in *NONO* transcript levels.

The phenotypic spectrum of MRXS34, now encompassing both sexes, is characterized by near-universal neurodevelopmental delay (GDD/ID), distinctive craniofacial features (macrocephaly, long face, micrognathia), and a high prevalence of cardiac defects (91.3%, LVNC being hallmark) (Table 1). Structural brain anomalies, particularly corpus callosum dysgenesis (85%), are highly characteristic. While our proband lacked overt callosal abnormalities, she exhibited white matter signal changes, suggesting a broader spectrum of neurological involvement. Hypotonia and feeding difficulties are common. Seizures occur but are less frequent (∼7%). Skeletal issues (scoliosis, joint laxity) and ophthalmological problems (strabismus) are also reported (19). Prenatal cases uniformly present with severe, often complex, cardiac malformations, frequently associated with other anomalies and poor outcomes (11, 16–20). Compared to typical severe male presentations, our female patient had relatively milder cardiac involvement (PFO vs. LVNC), potentially reflecting partial functional compensation from the residual wild-type allele expression on the extremely inactivated paternal X chromosome, particularly relevant in cardiac tissue. Alternatively, tissue-specific XCI patterns or modifier genes might attenuate cardiac severity in females, as suggested by studies on X-linked congenital heart disease contributions. However, the degree of neurodevelopmental impairment was substantial and comparable to males, suggesting the developing nervous system is exquisitely sensitive to *NONO* dosage.

The predominance of LOF variants (85.7%) scattered across *NONO* gene, underscores haploinsufficiency as the primary disease mechanism (Figure 4). Most LOF variants trigger NMD, leading to absent or severely reduced *NONO* protein, as demonstrated in patient fibroblasts (8). Compensatory upregulation of other DBHS proteins (SFPQ, PSPC1) occurs but is insufficient to rescue the phenotype (20) or potential structural disruption (e.g., disulfide bond formation interference) (13). The genotype-phenotype correlation remains complex; recurrent variants like p.Arg365* and p.Ala377 = (splicing defect) cause variable severity, likely influenced by genetic background and modifying factors (8, 21). ur novel variant (p.Gln322Lysfs*31) fits the LOF paradigm, causing NMD and protein truncation upstream of the critical coiled-coil domain. This case emphasizes that *NONO* screening is warranted not only in males with ID + cardiac defects (especially LVNC) +/- corpus callosum anomalies but also in females presenting with compatible phenotypes, necessitating adjunct XCI analysis.

The extreme XCI skewing (99:1) observed here is the key determinant enabling full phenotypic expression in our female patient. This mechanism parallels other X-linked disorders like Incontinentia Pigmenti or X-linked Lissencephaly in females (21). Skewed XCI is increasingly recognized as a diagnostic clue in unsolved neurodevelopmental cases, guiding re-evaluation of X-linked genes. Such skewing is common and influences disease severity in conditions like Fabry disease and various neurodevelopmental disorders (22). Additionally, XCI is often incomplete, with many genes “escaping” silencing, which creates natural differences in gene expression between males and females and contributes to individual variation (23). Crucially, testing for XCI skewing in undiagnosed patients—especially females with neurodevelopmental issues—is a powerful diagnostic tool, as it can reveal hidden disease-causing mutations on the X chromosome, significantly improving diagnostic rates (24). Studies on tissue-specific XCI landscapes further illustrate how inactivation patterns can modulate phenotypic severity across different organ systems (25, 26). Previously reported asymptomatic female carriers likely had random or favorable (wild-type active) XCI. The single prenatally diagnosed female fetus with a *NONO* VUS (p.Arg142His) highlights the diagnostic challenge and the need for functional studies and postnatal follow-up in such cases (27). Our patient's *de novo* frameshift variant (c.994del), coupled with 99% paternal X-chromosome inactivation, resulted in predominant mutant allele expression, overcoming typical X-linked protective mechanisms and manifesting as characteristic neurodevelopmental deficits alongside mild cardiac anomalies (patent foramen ovale). This parallels the pathophysiology of X-linked disorders like Incontinentia Pigmenti, emphasizing the diagnostic necessity of X-inactivation analysis in female evaluations (3). While the patient's cardiac phenotype was milder than typical male cases (possibly due to partial maternal X-chromosome compensation), the comparable severity of neurodevelopmental impairment suggests heightened nervous system sensitivity to *NONO* gene dosage effects. This case represents the first molecularly confirmed live-born female MRXS34 patient globally, contrasting with the single prior Chinese report (a male with p.Arg153* variant and asymptomatic female carriers) (9). By demonstrating how extreme XCI skewing can unmask X-linked recessive phenotypes in females, this case redefines the diagnostic paradigm for gender differences in X-linked disorders and significantly expands the clinical understanding of MRXS34.

For couples with a family history or previous affected child, genetic counseling should be offered, noting a 50% transmission risk to male offspring (who will be affected) and female offspring (who will typically be asymptomatic carriers). Prenatal diagnosis (via CVS/amniocentesis with WES or *NONO*-specific testing) and preimplantation genetic testing (PGT) are viable options, with prenatal ultrasound focusing on relative macrocephaly, cardiac defects (particularly LVNC, septal defects, and Ebstein anomaly), and corpus callosum abnormalities. Neonates/infants with suspected MRXS34 require immediate cardiac and respiratory evaluation due to risks of heart failure or pulmonary hypertension, alongside essential brain MRI and echocardiography (28). Early intervention for hypotonia, feeding difficulties (including potential NG/G-tube placement), and gastroesophageal reflux is crucial, as is prompt initiation of multidisciplinary rehabilitation (physical, occupational, and speech therapy). Long-term management involves regular monitoring of growth, nutrition, cardiac and neurological function, vision, hearing, and orthopedic status (e.g., scoliosis), with developmental assessments (e.g., Gesell, Bayley) guiding tailored educational and therapeutic support; anti-seizure medications may be necessary if epilepsy develops. Prognosis varies based on cardiac, neurological, and systemic involvement severity, with prenatal cases often having poor outcomes. While postnatal survival into adulthood is documented, significant neurodevelopmental disability is universal. The use of mouse nerve growth factor in our patient's rehabilitation regimen was based on its neurotrophic properties, which may support synaptic plasticity and neuronal survival in neurodevelopmental disorders; however, specific evidence in MRXS34 remains limited and warrants further investigation.

This study reports the first molecularly confirmed female case of MRXS34, caused by a *de novo NONO* frameshift variant (c.994del, p.Gln322Lysfs*31) and extreme skewing of X-chromosome inactivation (99%). This case definitively demonstrates that non-random XCI can unmask X-linked recessive disorders like MRXS34 in females, leading to phenotypes closely resembling those seen in affected males, albeit potentially milder in some systems (e.g., cardiac). Our findings significantly expand the clinical and genetic understanding of MRXS34, emphasizing the critical role of XCI analysis in the diagnostic workup of females with intellectual disability and compatible features. This case underscores the importance of including X-linked genes in the differential diagnosis for females with neurodevelopmental disorders and highlights *NONO* screening combined with XCI studies as a crucial diagnostic strategy for such patients. Future research should focus on elucidating tissue-specific effects of *NONO* haploinsufficiency, modifiers of XCI skewing, and potential therapeutic avenues targeting downstream pathways.

## Data Availability

The original contributions presented in the study are included in the article/Supplementary Material, further inquiries can be directed to the corresponding authors.
